# The Effects of Heat Shock Protein 70 Addition in the Culture Medium on the Development and Quality of In Vitro Produced Heat Shocked Bovine Embryos

**DOI:** 10.3390/ani11123347

**Published:** 2021-11-23

**Authors:** Konstantina Stamperna, Themistoklis Giannoulis, Eleni Dovolou, Maria Kalemkeridou, Ioannis Nanas, Katerina Dadouli, Katerina Moutou, Zissis Mamuris, Georgios S. Amiridis

**Affiliations:** 1 Department of Obstetrics and Reproduction, Veterinary Faculty, University of Thessaly, 431 00 Karditsa, Greece; konstantina.stamperna@gmail.com (K.S.); entovolou@uth.gr (E.D.); gnsnanas@gmail.com (I.N.); katerina1dad@gmail.com (K.D.); 2 Department of Animal Sciences, University of Thessaly, 413 36 Larissa, Greece; themisgia@gmail.com; 3 Laboratory of Genetics, Comparative and Evolutionary Biology, Department of Biochemistry and Biotechnology, University of Thessaly, 413 36 Larissa, Greece; mkalemkeridou@uth.gr (M.K.); kmoutou@uth.gr (K.M.); zmamur@uth.gr (Z.M.); 4 Laboratory of Hygiene and Epidemiology, Faculty of Medicine, University of Thessaly, 413 36 Larissa, Greece

**Keywords:** HSP70, heat stress, in vitro embryos, gene expression, cattle

## Abstract

**Simple Summary:**

European dairy cows are extremely sensitive to heat stress. During hot summers milk yield is suppressed, welfare status of the cows is aggravated, and their fertility is seriously compromised. The observed subfertility is attributed to endocrinological alterations and mainly to inability of the early embryo to develop uneventfully until the implantation stages. To protect the integrity and the functionality of their cells, all mammals have inherent defense mechanisms against external insults. A major representative of these molecules are the heat shock proteins (HSPs) that are secreted by almost all cells. In this study, we examined the effects of exogenous HSP70 on the developmental competence and quality of early embryos produced in vitro, after 24-h exposure to slightly elevated temperature. Our findings imply that the temperature rise is detrimental for the developmental potential of the early embryos, and that the HSP70 can partly mitigate the harmful effects of heat stress, by improving the embryo yield and by reducing the adverse effects of heat stress on some embryo quality characteristics.

**Abstract:**

The aims of the present study were to examine the effects of HSP70 addition in the in vitro culture medium of day 3 embryos on their developmental competence and quality. Bovine oocytes (*n* = 1442) were in vitro matured, inseminated and cultured for the first two days according to standardized methods. The presumptive zygotes were randomly allocated in three experimental groups: Control, C (embryos cultured at 39 °C throughout the culture period), group C41 (temperature was raised to 41 °C from the 48th to 72nd h post insemination (p.i.) and then it returned at 39 °C for the remaining culture period), and group H41 (the temperature modification was the same as in C41 and during heat exposure, HSP70 was added in the culture medium). Cleavage and embryo yield were assessed 48 h p.i. and on days 7, 8, 9, respectively and gene expression in day 7 blastocysts was assessed by RT-PCR. Blastocyst yield was the highest in group C39; and higher in group H41 compared to group C41. From the gene expression analyses, altered expression of 11 genes was detected among groups. The analysis of the orchestrated patterns of gene expression differed between groups. The results of this study confirm the devastating effects of heat stress on embryo development and provide evidence that HSP70 addition at the critical stages can partly counterbalance, without neutralizing, the negative effects of the heat insult on embryos, acting mainly through mechanisms related to energy deployment.

## 1. Introduction

Global warming, which is manifested with extended and hotter summers, undermines the sustainability and the profitability of the dairy sector, because during the long-lasting periods of high temperatures, the overall production cost is dramatically increased [1]. This is mainly contingent on the lower milk yield and mainly on the suppressed fertility. Using artificial cooling technologies in the summer, the losses in milk production are neutralized, but the fertility indices remain at alarming low levels [2]. The reduced fertility during the summer could be attributed to physiological alterations, such as hormonal imbalances that affect the developing follicle, the oviductal and the uterine environment, or to dysfunctions of cells that are directly involved in reproduction. The maturing oocyte is extremely sensitive to high temperatures. The follicle-enclosed maturing oocyte can be affected by the alterations occurring during heat stress in the follicular environment as high ambient temperatures increase the systemic FSH concentrations and decrease estradiol, inhibin and LH levels [3,4]. Exposure for 1 h of in vitro matured bovine oocytes to 41.5 °C does not affect blastocyst formation rate [5], but if the thermal insult (41 °C) lasts for 6 h [6] or 12 h [7] the embryo yield is seriously compromised, and it is further deteriorating if the maturing oocytes are exposed to 43 °C only for 45 min [5]. While the effects of heat stress on the maturing oocyte are undisputable, early embryos express a biphasic tolerance to heat stress being extremely vulnerable at early stages of development—until the 8-cell stage—, becoming more tolerant at later developmental stages. This is a long-known evidence for many species, such as cattle [8,9], sheep [10], rabbits [11] and swine [12], but the underlying mechanisms are not fully understood. Exposure to elevated temperatures of in vitro cultured bovine zygotes or 2-cell stage embryos, 4 to 8-cell stage embryos, or morulae results in developmental stage dependent reduction in blastocyst yield, being higher for zygotes or 2 cell stage embryos, intermediate for 4 to 8 cell embryos and slightly affected for morulae [7,13,14,15]. Chronically the acquisition of thermotolerance coincides with the 4th cell division (8 to 16-cell stage), at which embryonic genome activation occurs [16,17]. This stage is characterized by increased transcription activity [18], changes in protein synthesis [19] and functional organization of the nucleolus [20]. It is believed that after embryonic genome activation, most of the changes occurring at cellular functionality that make the embryo resilient to heat insults, include transcriptional activation of genes responsible for heat shock proteins [21]. A second, equally important defense mechanism of the embryo against the thermal insult is the antioxidant protection, which again in the early embryo is endogenously activated after the 8-cell stage [22].

Heat shock protein 70 (HSP70) is a molecular chaperon protecting almost all mammalian cells from many types of stress. In vertebrates, there are two main forms of HSP70s: several members exhibit constitutive expression patterns, while others are induced by various stresses that disrupt proper protein folding and they are regulated by HSF1. The evolutionary histories of HSP70s, and especially of the inducible forms, are quite complex with events of convergent evolution and gene duplication among others; hence, a new nomenclature for their classification has been proposed [23], Yu et al., 2021. The HSPs are closely associated with the cellular response to a variety of stressors, and they are not merely linked to the heat stress, as it was initially believed [24]. They are normally present in the cytoplasm, mitochondria, nucleus, endoplasmic reticulum, while during and after heat stress they localize mainly in the nucleus [25]. Under normal conditions, HSP70 participates in post translational folding and transportation of cellular proteins through the membranes [26,27], and it is now believed that it is involved in fertilization and early embryo development [28]. Addition of antibodies for HSP70 in early embryos cultured in vitro, suppresses the blastocyst formation rate both in cattle and mice [29,30]. Under heat stress, HSP70 plays a vital role by preserving the stability of the cytoskeleton, regulating the cell cycle and the immune response, preventing cell apoptosis, and contributing to the thermotolerance of cells [31,32]. During heat stress, HSPs can be found in the extracellular space [33], through mechanisms involving the lysosomes [34] via membrane-bounded particles [35] or by passive leaking from necrotic cells after the disruption of their membranes [33].

In a previous study we have shown that exposure of in vitro maturing bovine oocytes for only six h to elevated temperature (41 °C), causes reduced embryo development and induces perturbed expression of many genes [6]. This dramatic drop in embryo production and quality can be partly reversed with the addition of HSP70 in the maturation medium. Similarly, the addition of HSP70 altered the expression of genes related to metabolism, oxidation, apoptosis and thermotolerance in the cumulus cells and the early embryos [36]. The overarching goal of the present study was to examine whether the negative effects of exposing early bovine embryos to elevated temperature (41 °C) for 24 h could be mitigated by the addition of HSP70 into the culture medium.

## 2. Materials and Methods

### 2.1. In Vitro Embryo Production

All stages of the in vitro embryo production (IVP) were carried out according to the standardized laboratory protocols that have been previously described [36,37]. All chemicals were purchased from Sigma Chemical Company (Poole, UK), unless otherwise stated. Ovaries originating from Holstein cows were collected from neighboring abattoir and transported at 37 °C to the laboratory within 2 h from slaughter, in sterile saline (0.9% NaCl) with antibiotics (0.1% gentamycin). Cumulus oocyte complexes (COCs) were aspirated from 3–8 mm follicles by a hypodermic needle (18G) connected to 10mL disposable syringe. Selected grade 1 COCs [38] were first washed in PBS and then in maturation medium [TCM 199 supplemented with 10% fetal calf serum (FCS) and 10 ng/mL epidermal growth factor (EGF)]. In 7 experimental replicates 1442 COCs were matured for 24 h in an atmosphere of 5% CO_2_, 20% O_2_, with maximum humidity. In all groups in vitro maturation and fertilization were carried out under the same conditions, while in vitro culture conditions were modified as described below.

The matured COCs were inseminated with frozen-thawed, swim-up separated sperm, of the same ejaculation. The final sperm concentration used was 106 spermatozoa/mL. The gametes’ co-incubation lasted for 24 h in standard in vitro fertilization (IVF) medium at 39 °C, under an atmosphere of 5% CO_2_, 20% O_2_ with maximum humidity.

Twenty h after insemination (pi), the surrounding cumulus cells of the presumptive zygotes were removed by gentle vortexing and zygotes were cultured in groups of 25 in microdroplets (25 μL) under mineral oil. At this point the presumptive zygote of each replicate were randomly allocated in three experimental groups: Control, C (*n* = 396), C41 (*n* = 514), and HSP41 (*n* = 532). Zygotes were cultured for 9 days in synthetic oviductal fluid (SOF) supplemented with 5% FCS in an atmosphere of 5% CO_2_, 5% O_2_ and 90% N_2_, in maximum humidity. Control zygotes were incubated throughout the culture period in standard in vitro culture (IVC) medium at 39 °C. In groups C41 and HSP41 the zygotes were incubated for the first 24 h of culture at 39 °C; then the temperature was raised to 41 °C from the 25th until the 48th h of culture (72 h pi) and then it was switched to 39 °C for the remaining in vitro culture period. Group C41 embryos were cultured in standard IVC medium, while the group of H41 embryos were cultured in modified medium only for the period that they were incubated at 41 °C. This modification referred to the addition of HSP70 at a concentration of 5ng/mL. The dose was selected after the assessment of HSP70 concentrations in the peripheral blood of 164 heavily heat stressed Holstein cows [39]. To assure that the negative effect of manipulation of the embryos will not affect our results, the culture media of all groups were replaced according to the time schedule of group HSP41.

Cleavage and blastocyst formation rates were recorded under stereo-microscopic observation at 48h pi and on days 7, 8, and 9 pi, respectively. From 5 replicates, pools of 8 day 7 blastocysts were snap-frozen in PBS in liquid nitrogen and stored at −80 °C, until gene expression analysis.

### 2.2. RNA Extraction and Reverse Transcription

Total RNA was extracted using PicoPure^TM^ RNA Isolation Kit (Thermofisher Scientific, Waltham, MA, USA) according to the manufacturer’s protocol and was further treated with DNAfree^TM^ DNA Removal kit (Thermofisher Scientific, Waltham, MA, USA) to remove any DNA residuals. RNA’s quantity and quality were assessed using Qubit™ (Thermofisher Scientific, Waltham, MA, USA) RNA BR Assay Kit. The cDNA synthesis was performed using Maxima H Minus First Strand cDNA Synthesis Kit (Thermofisher Scientific, Waltham, MA, USA), 15 ng of total RNA and a combination of oligo-dTs and random primers. cDNA samples were further diluted (1:2 for the oocytes, 1:5 for the cumulus cells and the blastocysts) and were stored at −80 °C.

### 2.3. Gene Expression Analysis

To assess the effect of HSP addition in culture, a set of genes were selected, and their expression was quantified using real time PCR (qPCR). The gene expression of *HSF1*, *HSP90AA1*, *HSPA1A* (heat shock response), *GSTP1*, *GPX1* (antioxidant), *PLAC8A* (implantation), *DNMT3A* (epigenetic regulation), *ATPA1A* (osmoregulation), *IGF1*, *PTGS2* (cell signalling), *TLR2*, *TLR4*, *TNFA* (immunity), *AKR1B1* (metabolism), *BCL2* and *BAX1* (apoptosis) were analyzed.

qPCR was performed using the SYBR Green in an AB Step One Plus Mastercycler (Applied Biosystems). Analysis of qPCR was performed in a 20 μL reaction volume by adding 1.5 μL cDNA in the PCR mix containing gene specific primers (300 nM final concentration) and 1x KAPA SYBR FAST qPCR Master mix (Sigma-Aldrich). qPCR conditions were 5 min at 95 °C and 40 cycles of 20 s at 95 °C and 20 s at 60 °C for annealing and extension. At the end of every reaction, a melt curve analysis was performed to ensure the specificity of the products. Samples were measured in duplicates and a maximum ±0.2 difference in Cq values was applied as a threshold in the duplicates’ measurements. Primer pairs for each gene were designed using Primer-BLAST and Primer3. Primer pairs were further evaluated using Beacon Designer (http://www.premierbiosoft.com/qOligo/Oligo.jsp?PID=1, accessed on 4 July 2021) for their suitability (primer-dimers, hairpins’ formations etc.) and the UCSC In-Silico PCR tool to assess their specificity, using the latest available genome of the species (ARS-UCD1.2 in https://genome.ucsc.edu/cgi-bin/hgPcr, accessed on 4 July 2021). The complete list of primers is shown in Table 1.

The relative gene expression of all genes was normalized using three reference genes: *YWHAZ*, *UBA52* and *EEF1A1* (Table 1). The suitability of the reference genes was evaluated using GeNorm, and M value as an indicator of the gene expressions’ stability across samples. For the analysis of the differential gene expression between the groups (HSP addition versus control), we used a combinatorial LinReg-Quantification Cycle (Cq) approach: Cq values were retrieved for each reaction by setting a constant threshold and the average efficiencies per gene were computed using the LinReg software, as proposed by Ramakers and his colleagues [40]. The relative gene expression was normalized using the geometric mean of the three reference genes, as proposed by Vandesompele and colleagues [41].

### 2.4. Statistical Analyses

Statistical analyses on in vitro embryo production were carried out with IBM SPSS Statistics 25.0 for Windows. The results are expressed as means ± standard deviations. Data normality was checked using a Shapiro-Wilk test. Homogeneity of variances was assessed by Levene’s test of homogeneity of variance. Cleavage rates were tested between groups by one-way ANOVA. Multiple regression analysis was used to estimate the effect of Temperature and HSP70 addition on embryo yield between groups. Embryo development among groups C and C41 as well as C41 and H41 was tested by Student’s t-test. Significance was set at *p* ≤ 0.05.

The statistical analysis of differentially expressed genes (DEGs) was performed in R as follows: A Kruskal-Wallis test among the three groups was applied to detect the possible effect of HSP addition in gene expression (function kruskal.test), using a threshold of *p* ≤ 0.05 for significance.

A Dunn test was applied as an ad-hoc test, to detect pairwise differences between pairs of groups (C39 vs. C41, C39 vs. H41, C41 vs. H41), using a threshold of *p* ≤ 0.05 for significance.

Correlation coefficients were computed for each pair of genes in two groups using the rcorr function, since correlated gene expression may be indicative of a similar regulation mechanism underlying gene expression. Coefficients were plotted using the corrplot function, along with the corresponding *p*-values.

## 3. Results

### 3.1. In Vitro Embryo Development

The multiple regression analysis revealed that both variables i.e., temperature and HSP70 addition, predicted the embryo development rates on days 7 and 9 [day 7 F(2, 18) = 85.273, *p* < 0.001, R2 = 0.905; day 9 F(2, 18) = 43.082, *p* < 0.001, R2 = 0.827]. Both variables added significantly to the prediction (*p* < 0.05). On day 8 temperature embryo development rates was predicted (*p* < 0.05) by temperature but not by the HSP70 addition (F(2, 18) = 40.814, *p* < 0.001, R2 = 0.819), Table 2.

As expected, one-way ANOVA revealed no difference in cleavage rates between groups (*p* = 0.289). In group C39, blastocyst yield on days 7, 8, and 9 was steadily higher compared to the other groups, (*p* < 0.0001). In group H41 blastocyst yield was higher compared to that of group C41 on days 7 and 9 (*p* < 0.02 and *p* < 0.03, respectively) and strongly tended to be so on day 8 (*p* = 0.064). Details on embryo yield are given in Table 3.

### 3.2. Gene Expression

**Figure 1:**
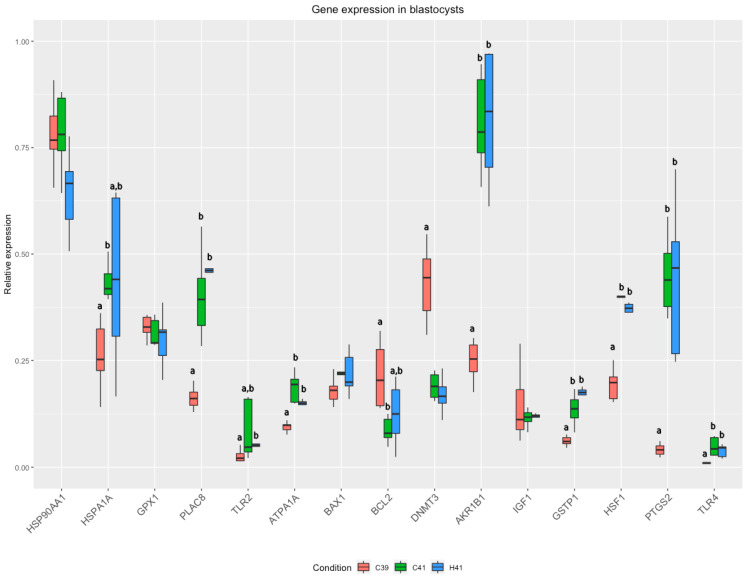
Gene expression in blastocysts cultured at 39 °C group (C39) or under raised temperature (41 °C) for 24 h in the absence (group C41) or the presence of HSP70 (group H41) in the culture medium. Within triplets, different letters denote significant cha changes (from the Kruskal-Wallis test and the ad-hoc Dunn test).

Statistical analysis revealed significant differences in the expression of 11 genes, including *HSPA1A*, *PLAC8*, *TLR2*, *ATPA1A*, *BCL2*, *DNMT3*, *AKR1B1*, *GSTP1*, *HSF1*, *PTGS2*, and *TLR4*. Pairwise significant differences are showed in Supplementary Table S1. Since we were mainly interested on the possible effects of HSP70 addition under heat stress conditions, we focused on differences between C41 and H41 groups. A strong tendency (*p* = 0.07) towards differential expression of 3 genes between C41 and H41 (*HSF1*, *HSP90AA1* and *ATP1A1*) was detected as well as a tendency towards significant differences in the expression of *TLR2* (*p* = 0.10) (Figure 1).

The C39 and the H41 group showed higher correlations in general in gene expression compared to the C41 group. In the C39 group, two groups of genes showed strong positive correlations: the first one included *IGF1*, *HSP90AA1*, *BCL2*, *TLR2* and the second one *ATP1A1*, *DNMT3*, *PLAC8*, *PTGS2*, *AKR1B1*, *TLR4*, *GSTP1* and *HSPA1A*.

**Figure 2:**
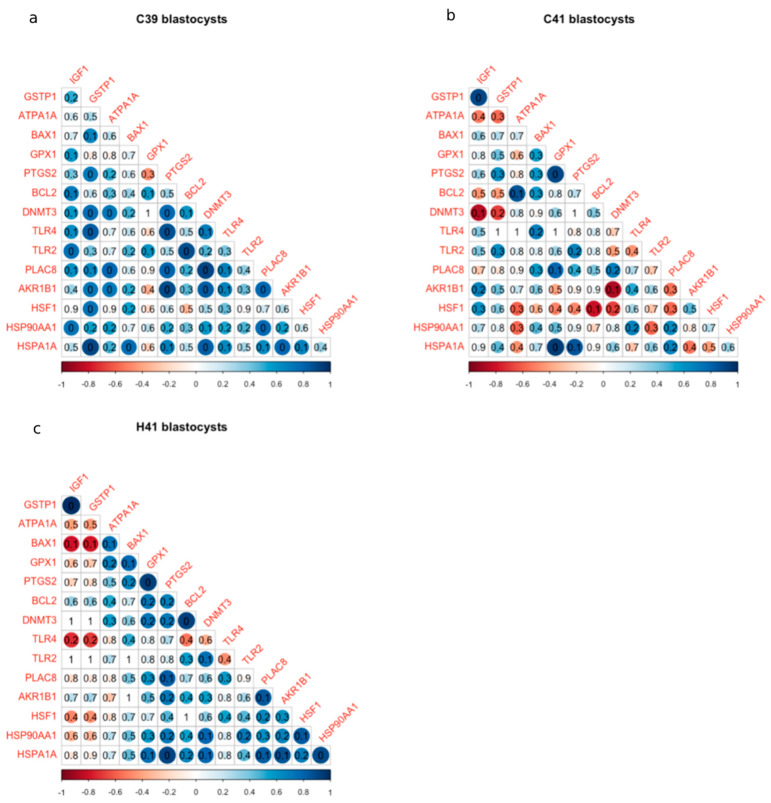
Pairwise correlation coefficients of genes in the groups under study: in blastocysts cultured at 39 °C group (C39, **a**) or under raised temperature (41 °C) for 24 h in the absence (group C41, **b**) or presence of HSP70 (group H41, **c**) in the culture medium. Positive correlations are displayed in blue and negative correlations in red color. Color intensity and the size of the circle are proportional to the correlation coefficients. *p*-values of each correlation are presented in each circle.

HSP70 supplementation led to high correlations (|r| > 0.5) in the expression of 6 genes (*ATPA1A*, *BAX1*, *GPX1*, *PTGS2*, *BLC2* and *DNMT3*) and between *PLAC8*, *AKR1B1*, *HSP90AA1*, *HSF1*, *HSPA1A*. Finally, *TLR4* and *BAX1* was negatively correlated with *IGF1* and *GSTP1*. In the C41 group, there were found sporadic correlations (positive or negative): *PTGS2* was positively correlated with *GPX1* and *HSPA1A*, while *DNMT3* was negatively correlated with *HSF1*, *AKR1B1*, *IGF1* and *GSTP1* and *ATPA1A* (Figure 2).

## 4. Discussion

In this study, embryos exposed to elevated temperature (41 °C) from 48–72 h of culture, expressed lower developmental capacity compared to the control counterparts. This finding agrees with a series of previous reports indicating the deleterious effects of heat stress on the developmental capacity of early cattle embryos [42,43,44,45]. Thermotolerance in the developing embryo seems to be associated with the embryonic genome activation (8-cell stage) [46], while the previous stages are exhibiting increased thermosensitivity [6,7,45,47]. In a previous study we have shown that the supplementation of in vitro maturation medium with HSP70 results in elevated thermotolerance capacity of oocytes under conditions of artificial heat stress and the effects are maintained at until the blastocysts stage [36]. Similarly, in the present study, the protective role of HSP70 was confirmed for the very early embryonic stages, as the supplementation of HSP70 in the heat stressed group showed significantly increased blastocysts yield compared to the heat stressed embryos without supplementation; however, the blastocysts yield was not restored at the levels of the C39 group. Although it was initially hypothesized that the 2-cell stage bovine embryos are sensitive to heat stress due to the lack or the minimal transcriptional activity, that is prerequisite to adapt to environmental challenges [48], it was soon shown that two cell embryos can respond to heat stress by synthesizing HSPs [49]. Opposite to the established belief, Lelièvre and co-workers [50] have shown that transcriptional response to severe or moderate heat stress can be observed only after the embryonic genome activation.

In our experiment the decrease in blastocyst development rates caused by the exposure to 41 °C for 24 h was more severe in the absence of HSP70 in the culture medium. It is well documented that HSP70 plays important role in fertilization and early embryo development [28]. Early murine or cattle embryos cultured in vitro in the presence of antibodies to HSP70 have reduced blastocyst formation and hatching rates [29,51]. HSP70 preserves the stability of the cytoskeleton, regulates the cell cycle and the immune response, prevents cell apoptosis, and contributes to the thermotolerance of cells [31,32]. HSP70 contributes to apoptosis blockade by interrupting the mechanism of caspase 3 activation [52], as well as by negatively regulating protein synthesis [32].

To the best of our knowledge, this is the first study to assess at molecular levels, the effects of HSP70 addition in the IVC medium on various gene expression in blastocysts. The temperature elevation (with or without HSP70 supplementation) lead to a series of differentiated regulations in gene expression, which were manifested by the changes in the relative expression of the genes (Figure 1), as well as by the changes in the coordinated patterns of gene expression (Figure 2). Eleven genes were found to have differential expression between the groups (Figure 1); these genes are involved in important cellular processes related to the response to stressors. *HSPA1A* encodes for an HSP70 protein, which acts as a molecular chaperon, mediating the proper folding of unfolded proteins and its expression was up regulated in bovine embryos under heat stress after the embryonic genome activation [50]. The expression of HSP genes is generally under the regulation of heat shock factors, encoded by HSF1 gene, which orchestrates the cascades of reactions under different types of stressors (heat stress, oxidative stress, etc.) [53]. The activation of heat shock response (HSR) and unfolded protein response (UPR) are responsible for the preservation of cellular homeostasis, mainly by refolding or degrading the misfolded proteins. However, the roles of HSF1 as a transcription factor is not limited in the HSP genes; it was also found that in human blood monocytes under heat stress HSF1 mediates the up regulation of expression of *PTGS2* [54]. The elevated levels of prostaglandins have been associated with negative effects on the development of cattle embryos, produced either in vitro or in vivo [55]; furthermore, elevated levels can cause premature luteolysis and subsequently embryo loss [56,57]. DNMT3 was found significantly lower in the heat stressed groups of embryos. This gene encodes for a DNA methyltransferase and it has been shown that its reduced expression has been associated with increased developmental capacity of the in vitro produced embryos [58,59]. On the other hand, we detected overexpression of *PLAC8* in C41 and H41 groups. *PLAC8* regulates placental development, and it is used as a marker of the embryo quality, since its higher expression has been linked with normal pregnancies and calving [60]. These significant changes in the expression of *PLAC8* and *DNMT3* under heat stress are in accordance with our previous results [6], where we evaluated the effects of heat stress imposed in oocytes on embryo yield and imply that embryos that can overcome the stressful conditions during their development are those having a resilient genotype for thermotolerance.

*TLR2* and *TLR4* encode for Toll-like receptors (TLRs), which are responsible for pathogen recognition and the initiation of the immune responses [61]. It has been shown that heat stress activates the TLR signaling pathways [62,63] and the upregulation detected in our study is in accordance with the results of Srikanth and colleagues [64], who, among others, reported the up regulation of *TLR2* and *TLR4* in Holstein calves under heat stress. The increased expression of TLRs appears to be associated with the increased expression of HSP70s (*HSPA1A* belongs to this family), which binds to *TLR2* and *TLR4*, inducing the immunoregulatory effects. However, the correlation of the gene expression shows a weak positive correlation in the C41 and H41 groups (Figure 2). *ATP1A1* encodes for one of the four subunits of Na +/K+ ATPase, which exchanges ions through the membrane, maintaining the proper electrochemical gradient [65] in an energy-dependent manner (approximately consuming 20–30% of total ATP in mammalian cells in rest [66]). The changes in Na+/K+ ATPase expression is responsible for cell membrane permeability alterations during heat stress [67], and it mediates HSP-mediated heat shock response by regulating the ATP balance [68]. It has been shown that *ATP1A1* expression is altered during heat stress in several cattle breeds [69,70,71]. The expression of *AKR1B1*, which protects against toxic aldehydes derived from lipid peroxidation, was increased during heat stress, along with the increase of HSP70, indicating the protective effects of HSP70 (exogenous and endogenous). This result is in accordance with our previous study [6]. *GSTP1* encodes for Glutathione S-Transferase Pi, which is implicated in the antioxidant mechanisms of the cells. Oxidative stress tends to increase under heat stress, resulting in elevated levels of ROS, negatively affecting the physiology and body metabolism [72]. The activation of the antioxidant mechanisms is essential for the cell survival, activating anti-apoptotic mechanisms [73].

By focusing on the differences between the heat stressed groups to evaluate the effect of HSP70 supplementation, we observed differences in the expression of three genes (*HSF1, HSP90AA1* and *ATP1A1*) that had a strong tendency towards significant downregulated in the H41 (Figure 1). *HSP90AA1* belongs to the family of HSP90 and encodes for a molecular chaperon, which participates in the proper folding of misfolded proteins using an ATPase activity; however, the protein is involved also in other functions, such as cell signalling, transcription and DNA replications and repair [74,75], yet they require excessive amounts of cellular energy to mitigate the negative effects of heat stress in cellular level. The roles of *HSF1* and *ATP1A1* are thoroughly discussed in the previous sections of the discussion.

In our belief, the supplementation of exogenous HSP70 in the embryo culture is acting protectively on multiple levels for the developing embryos: on one hand, the internalization of HSPs by the early embryos provides “ready-to-use” materials for the cell in order to cope with the unfolded proteins. On the other hand, it “relieves” the embryos from the need to express specific genes (like HSFs, HSPs, etc.) and to activate energy demanding processes and cascades, namely the UPR and HSR, to mitigate the undesired effects of heat stress. In this way we infer that the embryos are not energetically exhausted and can allocate the excess of energy in other processes, such as cell mitoses and genome activation that are essential for their development.

Comparing the orchestrated patterns of gene expression (Figure 2), our major finding was the disruption of coordinated expression of genes in the C41 group. On the other hand, C39 group showed a tight regulation in the expression of several genes related to the developmental capacity and the quality of the embryo, as well as, in the regulation of protective mechanisms. *GSTP1, BAX1, PTGS2, DNMT3, TLR4, PLAC8, AKR1B1, HSF1* and *HSPA1A* exhibited strong and positive pairwise correlations, which is indicative of their coordinated pattern of expression. This coordination appears to be essential to support proper development and quality of the embryo under normal conditions. *GSTP1*, *HSF1*, *HSPA1A* and *BAX1* participate in the defense mechanisms against stressors (oxidative and heat stress) to protect the cells from apoptosis. *HSF1* appears to be a link between the common regulations of these procedures [53]. However, under heat stress conditions (C41 group), the tight coordination of the expression of these genes seems to be non existing and strong correlation patterns were scarce. *HSPA1A* was strongly correlated with *GPX1* and *PTGS2*, which, as mentioned above, reflects the tight regulation of HSP70 with antioxidant genes and prostaglandin production. It is noteworthy that the exogenous HSP70 supplementation restored the coordinated gene expression of most of the genes; yet the new gene networks included different combinations of the genes compared to C39. *HSF1*, *HSP90AA1* and *HSPA1A* showed positive correlation along with *AKR1B1* and *PLAC8*, which links the protective mechanisms with the developmental potential of the embryos. On the other hand, *DNMT3, BCL2, PTGS2, GPX1, BAX1* and *ATP1A1* were strongly correlated; *GPX1*, *BCL2* and *BAX1* are apoptosis regulators, while *ATP1A1* and *PTGS2* expression is commonly affected by heat stress as mentioned above.

In conclusion, it was demonstrated that the external supplementation of HSP70 can partly offset the deleterious effects of heat stress in blastocysts formation. We hypothesize that this was achieved by enhanced provision of essential proteins for heat shock response, and by increasing energy availability that is required for the consequent embryo development.

## Figures and Tables

**Table 1:** Primer information: sequence, size of the amplified fragments of transcripts and accession number.

Gene Name	Accession Number	Gene Description	Forward Primer	Reverse Primer	Product Size (bp)
EEF1A1	ENSBTAG00000014534	Eukaryotic translation elongation factor 1 alpha 1	CCCCAGGACACAGAGACTTC	ATTCACCAACACCAGCAGCA	93
YWHAZ	ENSBTAG00000000236	Tyrosine 3-monooxygenase/tryptophan 5-monooxygenase activation protein zeta	CTGTAACTGAGCAAGGAGC	CCAAGATGACCTACGGGC	95
UBA52	ENSBTAG00000007737	Ubiquitin A-52 residue ribosomal protein fusion product 1	CCGCAAGAAGAAGTGTGGC	GCAAAGGAGAAGCAGGTGGA	84
HSPA1A	ENSBTAG00000025441	Heat shock protein family A (Hsp70) member 1A	GGACCTGCTGTTGCTGGAC	TTCGTGGGGATGGTGGAGTT	102
HSP90AA1	ENSBTAG00000006270	Heat shock protein 90 alpha family class A member 1	CTGGAAGGAGACGACGACAC	ACACACTGGAGGGAATGGAG	104
GPX1	ENSBTAG00000054195	Glutathione peroxidase 1	GAAAAGTGCGAGGTGAATGG	GAGAGCAGTGGCGTCGTC	93
PLAC8	ENSBTAG00000009849	Placenta specific 8 A	GTTTCACAGCCAGGTTACAGC	AGAGCCCCACAGAGACAGAT	104
TLR2	ENSBTAG00000008008	Toll like receptor 2	GCTGCCATTCTGATTCTGCT	GCCACTCCAGGTAGGTCTTG	103
ATP1A1	ENSBTAG00000001246	ATPase Na +/K+ transporting subunit alpha 1	CGCCAGGGTTTATCCAGTT	AGGGGAAGCCAGTTTTTGTT	80
BAX	ENSBTAG00000013340	BCL2 associated X, apoptosis regulator	TTTGCTTCAGGGTTTCATCC	CGCTTCAGACACTCGCTCAG	120
BCL2	ENSBTAG00000019302	BCL2 apoptosis regulator	CCCTGTTTGATTTCTCCTGGC	CTGTGGGCTTCACTTATGGC	107
DNMT3A	ENSBTAG00000021143	DNA methyltransferase 3 alpha	GAAGGAGCATTTGGGAACAG	GTTATTGCGTGAGCCTGGAT	118
AKR1B1	ENSBTAG00000009902	Aldo-keto reductase family 1, member B1 (aldose reductase)	GAAAGTGGTGAAGCGTGAGG	TAGAGGTCCAGGTAGTCCAGC	129
IGF1	ENSBTAG00000011082	Insulin like growth factor 1	TCACATCCTCCTCGCATCTCTT	AGCATCCACCAACTCAGCC	107
GSTP1	ENSBTAG00000003548	Glutathione S-transferase pi 1	TGGAAGGAGGAGGTGGTGAC	CAGGTGACGCAGGATGGTATTG	211
HSF1	ENSBTAG00000020751	Heat shock transcription factor 1	ATGAAGCACGAGAACGAGGC	GCACCAGCGAGATGAGGAACT	112
PTGS2	ENSBTAG00000014127	Prostaglandin-endoperoxide synthase 2	AGTCTTTGGTCTGGTGCCTG	AACAACTGCTCATCGCCCC	117
TLR4	ENSBTAG00000006240	Toll like receptor 4	AGGTAGCCCAGACAGCATTT	GAGCGAGTGGAGTGGTTCA	110

**Table 2:** Effects of Temperature during in vitro culture and HSP70 addition in the IMC medium on blastocyst formation rates on days 7, 8 and 9.

Stage of Embryos	Factor	B- Coefficients with 95% CI	Sig.
Day-7 blastocysts	Temperature	−9.1 (−10.7, −7.6)	<0.001
	HSP70	3.9 (0.8, 6.9)	0.017
Day-8 blastocysts	Temperature	−10.0 (−12.5, −7.5)	<0.001
	HSP70	4.1 (−0.8, 9.0)	0.096
Day-9 blastocysts	Temperature	−9.9 (−12.2, −7.5)	<0.001
	HSP70	4.8 (0.2, 9.5)	0.043

Multiple regression (Dependent variable: embryo development rates on days 7, 8, 9, respectively and independent variables: Temperature and HSP70).

**Table 3:** Blastocyst formation rates in control embryos (group C), embryos exposed to elevated temperature for 24 h (C41) and embryos exposed to elevated temperature with the addition of HSP70 in the IVC medium (group H41).

Group	Presumptive Zygotes	Cleavage (%)	Day-7 Blastocysts (%)	Day-8 Blastocysts (%)	Day-9 Blastocysts (%)
C	396	327 (82.6 ± 2.3)	108 (27.3 ± 2.1) ^a^	137 (34.6 ± 5.4) ^a^	146 (36.9 ± 5.1) ^a^
H41	532	427 (80.2 ± 2.1)	68 (12.8 ± 2.0) ^b^	96 (18.0 ± 3.2) ^b^	114 (21.4 ± 3.5) ^b^
C41	514	411 (80.3 ± 3.5)	47 (8.9 ± 3.8) ^c^	70 (13.6 ± 4.3) ^b^	82 (15.9 ± 3.6) ^c^

Within rows, values marked with different superscripts differ significantly.

## Data Availability

The data presented in this study are available from the corresponding author upon reasonable request.

## Supplementary Materials

The following are available online at https://www.mdpi.com/article/10.3390/ani11123347/s1, Table S1: Pairwise significant differences in gene expression between groups of embryos cultured in standard media and temperature (group C39), in standard medium with elevated temperature for 24 h (group C41) and in modified medium with the addition of HSP70 with temperature elevation for 24 h (group H41).
