# Supplementary material for: The Effects of Heat Shock Protein 70 Addition in the Culture Medium on the Development and Quality of In Vitro Produced Heat Shocked Bovine Embryos
Source: Animals (Basel). 2021 Nov 23;11(12):3347. doi: 10.3390/ani11123347 (PMC8698181; doi:10.3390/ani11123347)
Supplement: Supplementary file 1 [file animals-11-03347-s001.zip › animals-1469748-supplementary.pdf]

Supplementary Table S1: Pairwise significant differences in gene expression between groups of embryos cultured in standard media and temperature (group C39), in standard medium with elevated temperature for 24 hours (group C41) and in modified medium with the addition of HSP70 with temperature elevation for 24 hours (group H41).

Gene	C39 - C41	C39 - H41	C41 - H41
**HSP90AA1**	-	-	±
**HSPA1A**	*	-	-
**GPX1**	-	-	-
**PLAC8**	*	*	-
**TLR2**	-	*	-
**ATPA1A**	*	*	±
**BAX1**	-	-	-
**BCL2**	-	*	-
**DNMT3**	*	*	-
**AKR1B1**	*	*	-
**IGF1**	-	-	-
**GSTP1**	*	*	-
**HSF1**	*	*	±
**PTGS2**	*	*	-

Significant differences (p-adjusted < 0.05) are marked with an asterisk. The three comparisons marked with ± symbol tended to differ significantly (p=0.07).
